# Non‐invasive treatment of ischemia/reperfusion injury: Effective transmission of therapeutic near‐infrared light into the human brain through soft skin‐conforming silicone waveguides

**DOI:** 10.1002/btm2.10496

**Published:** 2023-02-07

**Authors:** Paul T. Morse, Samuel Tuck, Mike Kerns, Dennis J. Goebel, Junmei Wan, Tom Waddell, Joseph M. Wider, Charlotte L. Hüttemann, Moh H. Malek, Icksoo Lee, Thomas H. Sanderson, Maik Hüttemann

**Affiliations:** ^1^ Center for Molecular Medicine and Genetics Wayne State University School of Medicine Detroit Michigan USA; ^2^ Department of Emergency Medicine University of Michigan Medical School Ann Arbor Michigan USA; ^3^ Lumitex, Inc. Strongsville Ohio USA; ^4^ Department of Ophthalmology, Visual and Anatomical Sciences Wayne State University School of Medicine Detroit Michigan USA; ^5^ Mitovation, Inc. Innovation Partnerships Startup Incubator University of Michigan North Campus Research Complex Ann Arbor Michigan USA; ^6^ Department of Health Care Sciences Eugene Applebaum College of Pharmacy & Health Sciences, Wayne State University Detroit Michigan USA; ^7^ College of Medicine Dankook University Cheonan‐si Chungcheongnam‐do Republic of Korea; ^8^ Department of Biochemistry, Microbiology and Immunology Wayne State University Detroit Michigan USA

**Keywords:** cadaver, infrared light, ischemia/reperfusion, light penetration, mitochondria, stroke, waveguide

## Abstract

Noninvasive delivery of near‐infrared light (IRL) to human tissues has been researched as a treatment for several acute and chronic disease conditions. We recently showed that use of specific IRL wavelengths, which inhibit the mitochondrial enzyme cytochrome *c* oxidase (COX), leads to robust neuroprotection in animal models of focal and global brain ischemia/reperfusion injury. These life‐threatening conditions can be caused by an ischemic stroke or cardiac arrest, respectively, two leading causes of death. To translate IRL therapy into the clinic an effective technology must be developed that allows efficient delivery of IRL to the brain while addressing potential safety concerns. Here, we introduce IRL delivery waveguides (IDWs) which meet these demands. We employ a low‐durometer silicone that comfortably conforms to the shape of the head, avoiding pressure points. Furthermore, instead of using focal IRL delivery points via fiberoptic cables, lasers, or light‐emitting diodes, the distribution of the IRL across the entire area of the IDW allows uniform IRL delivery through the skin and into the brain, preventing “hot spots” and thus skin burns. The IRL delivery waveguides have unique design features, including optimized IRL extraction step numbers and angles and a protective housing. The design can be scaled to fit various treatment areas, providing a novel IRL delivery interface platform. Using fresh (unfixed) human cadavers and isolated cadaver tissues, we tested transmission of IRL via IDWs in comparison to laser beam application with fiberoptic cables. Using the same IRL output energies IDWs performed superior in comparison to the fiberoptic delivery, leading to an up to 95% and 81% increased IRL transmission for 750 and 940 nm IRL, respectively, analyzed at a depth of 4 cm into the human head. We discuss the unique safety features and potential further improvements of the IDWs for future clinical implementation.

**Abbreviations**
ABS: acrylonitrile butadiene styrene
CNC: computer numerical control
COX: cytochrome *c* oxidase
ETC: electron transport chain
FBT: fused biconical taper
FC: ferrule connector
FDM: fused deposition modeling
IDW: IRL delivery waveguide
IRL: near‐infrared light
NA: numerical aperture
PMMA: polymethyl methacrylate
ROS: reactive oxygen species
SMA: sub‐miniature A connector
SPU: surface percentage uniformity
ΔΨ_m_
: mitochondrial membrane potential

## 1 INTRODUCTION

Stroke is one of the leading causes of death globally, with more than 15 million people affected each year.^1^, ^2^ With the blood flow to the tissue blocked due to the stroke, the affected area quickly becomes ischemic. During ischemia, energy is rapidly depleted due to the lack of oxygen preventing oxidative phosphorylation by the mitochondrial electron transport chain (ETC).^3^ Disrupted mitochondrial calcium levels and the low ATP concentration result in activation of the various mitochondrial complexes in a futile attempt to meet the energy demand.^4^, ^5^ The restoration of blood flow is necessary to prevent total death of the affected area, but also results in transient mitochondrial hyperpolarization and excessive reactive oxygen species (ROS) production, which causes reperfusion injury.^6^, ^7^

Clinical trials to treat stroke pharmacologically, such as with ROS scavengers, have failed to deliver clinically in human patients despite success in the laboratory environment with animal models.^8^, ^9^ These failures may be due, in part, to difficulties in delivering relevant concentrations to the specific subcellular targets during the early period of reperfusion, when most reactive oxygen species (ROS) are generated. The spectrum of near‐infrared light (IRL), ranging from 700 to 1000 nm, has been evaluated as a possible noninvasive treatment for stroke.^10^ IRL offers many benefits over conventional treatments. IRL is nonpharmacological and therefore can be noninvasively applied locally and does not affect the body systemically. Additionally, IRL does not depend on blood flow, which is compromised during stroke, to be delivered to the target tissue.

Cytochrome *c* oxidase (COX), also known as complex IV in the mitochondrial ETC, has been identified as the primary target of IRL due to its two enzymatic copper centers, which broadly absorb IRL between 700 and 1000 nm as can be seen with the COX absorption spectrum.^11^, ^12^, ^13^ Although the precise mechanism of IRL action on COX is not fully clear, it should be noted that there are two copper atoms located at the cytochrome *c* binding site (electron acceptor site) and a third copper atom is located near heme *a*_3_ and binds the COX substrate, oxygen. Therefore, all three coppers are located at the catalytic sites of COX. Since infrared light adds vibrational energy when absorbed, a straightforward hypothesis would be that additional movement at the catalytic site(s) alters cytochrome *c* and/or oxygen binding. Generally, IRL is believed to be stimulatory to COX and the mitochondria, particularly the commonly used 810 nm.^14^ Treatment strategies with 810 nm focus on increasing mitochondrial activity in areas affected by stroke to increase ATP levels and improve cognitive function.^10^ However, we have previously established that certain wavelengths of IRL, specifically 750 nm and 940/950 nm, are inhibitory to COX and the mitochondria.^15^ This provides a unique treatment opportunity for reperfusion injury following ischemia. By applying COX‐inhibitory IRL during early reperfusion, ETC flux is reduced in the hyperpolarized mitochondria, which normalizes the mitochondrial membrane potential and prevents the generation of excessive ROS. We have demonstrated inhibition using both purified COX protein and intact cells. Additionally, applying 750 and 950 nm during early reperfusion showed robust neuroprotection in rat models of focal stroke and global brain ischemia.^15^, ^16^

The IRL spectrum is able to penetrate through biological tissues via an “optical window” which occurs due to the low absorbance of hemoglobin above 650 nm and water below 1000 nm.^17^ Despite this, most IRL is lost within the first millimeter of the skin.^18^ This highlights the need for the development of IRL delivery technologies that can enhance transmission of IRL through biological tissues to reach therapeutic amounts of IRL at the deepest structures of the brain. Here, we describe the development and testing of our IRL delivery waveguides (IDWs) which facilitate the transmission of IRL into the brain. Our IDWs consist of low‐durometer silicone waveguides, which have good optical transparency in the near IRL range.^19^ Additionally, silicone waveguides have previously been used to efficiently transmit IRL for spectroscopic purposes and biomarker detection.^20^, ^21^, ^22^ Similar technology has been found to enhance delivery of light to a target tissue.^23^

Our IDWs offer several advantages over traditional IRL delivery methods. The IDWs are placed in direct contact with the scalp, conforming to the patient's head to prevent pressure points. IRL is transmitted through fiberoptic cables from a laser source to the IDWs, allowing higher IRL outputs without the heat production associated with light‐emitting diodes (LEDs).^24^ Extract steps within the IDWs allow uniform distribution of IRL toward the scalp, preventing “hot spots” which could burn the skin. Additionally, the rear surface of the IDW is coated with a reflective material to capture IRL that reflects off the skin and redirect it back toward the body. Furthermore, this solution is scalable, providing a novel platform for IRL delivery into any desired tissue type. We have previously reported that COX‐inhibitory IRL can penetrate up to 4 cm into the human head using fresh, unfixed cadavers.^25^ Here, using the same wavelengths and IRL output intensities, we compare IRL penetration via fiberoptic delivery of IRL versus IRL delivered by our IDW into the human head and isolated human skin samples.

## 2 MATERIALS AND METHODS

### 2.1 Waveguide design

The waveguide form was designed by Mitovation, Inc. (Ann Arbor, MI) and Lumitex, Inc. (Strongsville, OH) and optimized by Lumitex, Inc. with consultation from Gray Optics (Portland, ME). Monte Carlo simulations of IRL ray traces were run through a virtual waveguide model using Zemax OpticStudio® software to define the extraction feature and IRL‐receiving lens geometries that satisfy the waveguide performance criteria. The waveguide solid body was designed using SOLIDWORKS® 2020 computer‐aided design software. Waveguide models were computer numerical control (CNC) milled from polymethyl methacrylate (PMMA) by ProtoLabs (Maple Plain, MN) to verify that the simulation data accurately predicted the physical model's performance. The final waveguide form was injection molded from Nusil MED‐6033 clear low‐consistency silicone elastomer by ProMed (Minneapolis, MN).

### 2.2 IDW assembly and system integration

The IRL delivery unit clamshell housing was CNC milled from polyoxymethylene (Delrin®) by ProtoLabs. The waveguide‐clamshell housing assemblies were secured with number 0 size thread‐forming screws from McMaster‐Carr (Elmhurst, IL). Two (one for each laser diode) 1 × 7 multimode fused biconical taper (FBT) fiberoptic splitters, sourced from LFiber Optic Limited (Shenzhen, Guangdong, China), were customized with a 400 μm core with a 0.22 numerical aperture (NA) step index, a sub‐miniature A (SMA) 905 threaded connector on the IRL input fiber, and 2.5 mm diameter ferrule connectors (FC) on each of the seven output fiber tips. Two SMA‐to‐SMA mating sleeves purchased from ThorLabs (Newton, NJ) were used to connect each laser diode to a splitter aligning the laser diode fiber guide ferrule and splitter input fiber guide ferrule apertures tip‐to‐tip. A custom IDW‐splitter fixture assembly was 3D‐printed by ProtoLabs from acrylonitrile butadiene styrene (ABS)‐like gray photopolymer resin using a stereolithography printer. Each IDW assembly received three of the fibers from the splitter connected to the 750 nm laser diode assembly from Akela Laser (Jamesburg, NJ) and three fibers from the splitter connected to the 940 nm laser diode assembly from BWT Beijing Ltd. (Fengtai, Beijing, China) leaving a null fiber (center fiber) disconnected from each splitter for monitoring output stability from each laser diode when necessary.

### 2.3 IDW performance verification

A performance verification study was conducted to evaluate the optical delivery performance of the IDWs (*n* = 5 IDWs). Each IDW assembly was evaluated for extraction efficiency and uniformity of IRL power across the emitting surface.

### 2.4 Extraction efficiency

The waveguide emitting surface contributing output power from each laser diode was measured using a 5 cm diameter Thermopile Sensor and an Optical Laser Power Meter, model 843‐R, from Newport (Avenue Irvine, CA). To determine the power contribution from each wavelength separately, the corresponding wavelength was selected in the handheld optical meter menu. Power (W) from the splitter fibers connected to the 750 nm laser diode $\left(P_{750\text{in}}\right)$ and 940 nm laser diode $\left(P_{940\text{in}}\right)$ was measured by first offsetting the ambient light with the meter's “Zero” function before deploying the laser. The laser diode's power supply was incrementally adjusted until the desired output from the three contributing fibers was achieved for each wavelength contributing power measurement. The sum of the contributing power inputs from each wavelength was the total power input ($T_{\text{in}}$) to one IDW:

(1) $$\text{Total Input Power}\;\left(T_{\text{in}}\right)=P_{750\text{in}}+P_{940\text{in}}\;\left(W\right)$$

This process was repeated using the IDW as an extended IRL delivery source after placing three output ferrules from the 750 nm splitter and three ferrules from the 940 nm splitter into each of the IDW housing's six input dowel holes in alternating sequence. Before deploying the laser diode, the waveguide emitting surface was placed over the 5 cm sensing aperture of the thermopile and the ambient light was offset with the “Zero” function. The sum of the contributing power outputs from each wavelength was the total power output ($T_{\text{out}}$) of one IDW:

(2) $$\text{Total Out Power}\;\left(T_{\text{out}}\right)=P_{750\text{out}}+P_{940\text{out}}\left(W\right)$$

The IDW extraction efficiency was measured for each wavelength by dividing the total power output from the waveguide emitting surface ($T_{\text{out}}$) by the total power input of IRL from the splitter ($T_{\text{in}}$).

(3) $$\%\text{Total Extraction Efficiency}=\left[\frac{T_{\text{out}}\;}{T_{\text{in}}\;}\right]\times 100$$

### 2.5 Uniformity pixel analysis and surface percentage uniformity

To quantitatively verify the distribution of output IRL power across the waveguide's emitting surface, six inputs ferrules from the splitter connected to the 940 nm IRL source were connected to the IDW. The output power from the 940 nm IRL source was incrementally adjusted until the total measured output from the emitting surface was 2 W using the methods described above. A photomask was 3D‐printed from Nylon by ProtoLabs using fused deposition modeling (FDM) printing. The photomask dimensions were designed to fit the dimensions of an 818P Series High Power Detector connected to an 842‐PE series Handheld Power/Energy Meter from Newport. The top face of the photomask contained a grid of 4 × 4 mm squares with one square cut‐out in the middle of the grid allowing IRL to pass through to the sensing aperture. The distal‐left corner of the waveguide emitting surface was placed over the square cut out such that the edges were aligned. The ambient light was offset using the previously described procedure before the IRL was deployed. The IRL power was measured at the first location after on the emitting surface through the 4 × 4 mm pixel allowing the sensor to stabilize for 20 s and repeated by moving the waveguide along the grid until power from each of the 25 pixel locations along the 4 cm^2^ emitting surface was recorded. The pixels with the maximum power (${\text{Power}}_{\max})$ and the pixels with the minimum power (${\text{Power}}_{\min}$) were recorded. The surface percentage uniformity (SPU) was calculated using the following equation:

(4) $$\mathrm{SPU}=\left\{1-\left[\frac{{\text{Power}}_{\max}-{\text{Power}}_{\min}}{{\text{Power}}_{\max}+{\text{Power}}_{\min}}\right]\right\}\times 100$$

### 2.6 Emitting surface imaging

The emitting surfaces of IDWs (*n* = 5) were imaged using a D5500 Nikon DSLR camera that was modified to capture IRL. Six inputs ferrules from the splitter connected to the 940 nm IRL source were connected to the IDW. The output power from the 940 nm IRL source was incrementally adjusted until the total measured output from the emitting surface was 2 W using the methods described above. The camera was fitted with a Nikon AF‐S VR Micro‐NIKKOR 105 mm *f*/2.8G IF‐ED lens containing a Hoya R72 infrared filter. A piece of sand‐blasted glass (Edmund Optics, Barrington, NJ) was used as a diffuser and positioned 20 cm away from the camera lens. The IDW emitting surface was positioned directly behind the diffuser, parallel to the glass surface, and aligned within the field of view using the gantry's adjustable axes. Captured images of the emitting surface were uploaded into ImageJ and changed to an 8‐bit gray scaled image. The line tool was used to trace the edge of the emitting surface and create a scaled conversion from pixel to mm. The rectangle tool was used to select the region of interest across the emitting surface. The “Plot Profile” tool was used to plot the combined average gray value intensity of pixels in the Y‐axis against its location along the X‐axis. The image was rotated 90 degrees and this process was repeated to plot the intensity along the perpendicular axis.

### 2.7 Cadaver donation

A cadaver was made available for research by the Body Bequest program at Wayne State University School of Medicine, Department of Ophthalmology, Visual and Anatomical Sciences. In accordance with the Uniform Anatomical Gift Act of Michigan (Act. No. 368, Public Acts of 1978, Article 10), donor consent for educational and research purposes was obtained prior to death. Post‐mortem, the cadaver was transported to Wayne State University and stored at 4°C. Surgical dissection and IRL transmission experiments were performed at 16°C.

### 2.8 Surgical dissection

Surgical dissection of the cadaver proceeded as previously described.^25^ After shaving the head, skin and soft tissues of the scalp were excised to expose the skull. An arch‐shaped window into the brain was generated via craniotomy of the skull using a Stryker 810 Autopsy Saw (Kalamazoo, MI). Lastly, the ThorLabs PM160 optical power meter (Newton, NJ) was inserted into the brain after performing a micro‐durotomy at the site, and IRL transmission measurements were taken. After IRL transmission measurements on the intact head were complete, an isolated skin sample was taken from the top of the scalp.

### 2.9 IRL transmission measurements through cadaver head

After the dissection, the optical power meter was sequentially inserted into occipital, parietal, frontal, and temporal lobes of the brain. For the duration of the experiment, the Thor PM160 optical power meter was covered with SC Johnson Ziploc brand snack bags (Racine, WI) cut and heat sealed to the dimensions of the optical power meter to prevent biological matter from directly contacting the detector surface. This barrier consists of transparent plastic material that did not absorb IRL during testing. After insertion of the power meter into each lobe at a distance of 4 cm from the skin of the head using a stereotactic tool,^25^ 750 and 940 nm IRL transmission measurements were taken using a 4 W power input emitted from the laser‐connected fiberoptic cable located 2 cm away from the surface of the skin. Prior to changing lobes, the laser was replaced with the waveguide. Then, 750 and 940 nm IRL transmission measurements were repeated using a 4 W power output emitted from the waveguide making direct contact with the surface of the skin. Background measurements with no IRL emission were recorded for both the laser and waveguide. The experimental transmission measurements were taken by operating the laser diodes continuously.

### 2.10 IRL transmission measurements through isolated cadaver skin

An isolated skin sample from the cadaver was taken after IRL transmission measurements were performed on the head. IRL transmission measurements on the skin sample were performed with an Optical Power/Energy Meter, model 842‐PE, from Newport. For the duration of the experiment, the optical power meter was covered with CVS Pharmacy Total Home Stretch‐Tite Plastic Wrap (Woonsocket, Rhode Island) to prevent biological matter from directly contacting the detector surface. This barrier consists of transparent plastic material that did not absorb IRL during testing. As previously described, the skin sample was processed and placed onto the detector surface.^25^ The 750 and 940 nm IRL transmission measurements were taken using a 1 W power output emitted from the laser‐connected fiberoptic cable located 3 cm away from the surface of the detector. Then, 750 and 940 nm IRL transmission measurements were repeated using a 1 W power output emitted from the waveguide making direct contact with the surface of the skin. Skin sample was 0.37 cm thick. Background measurements with no IRL emission were recorded for both the laser and waveguide. The experimental transmission measurements were taken by operating the diodes continuously.

## 3 RESULTS

**FIGURE 1:**
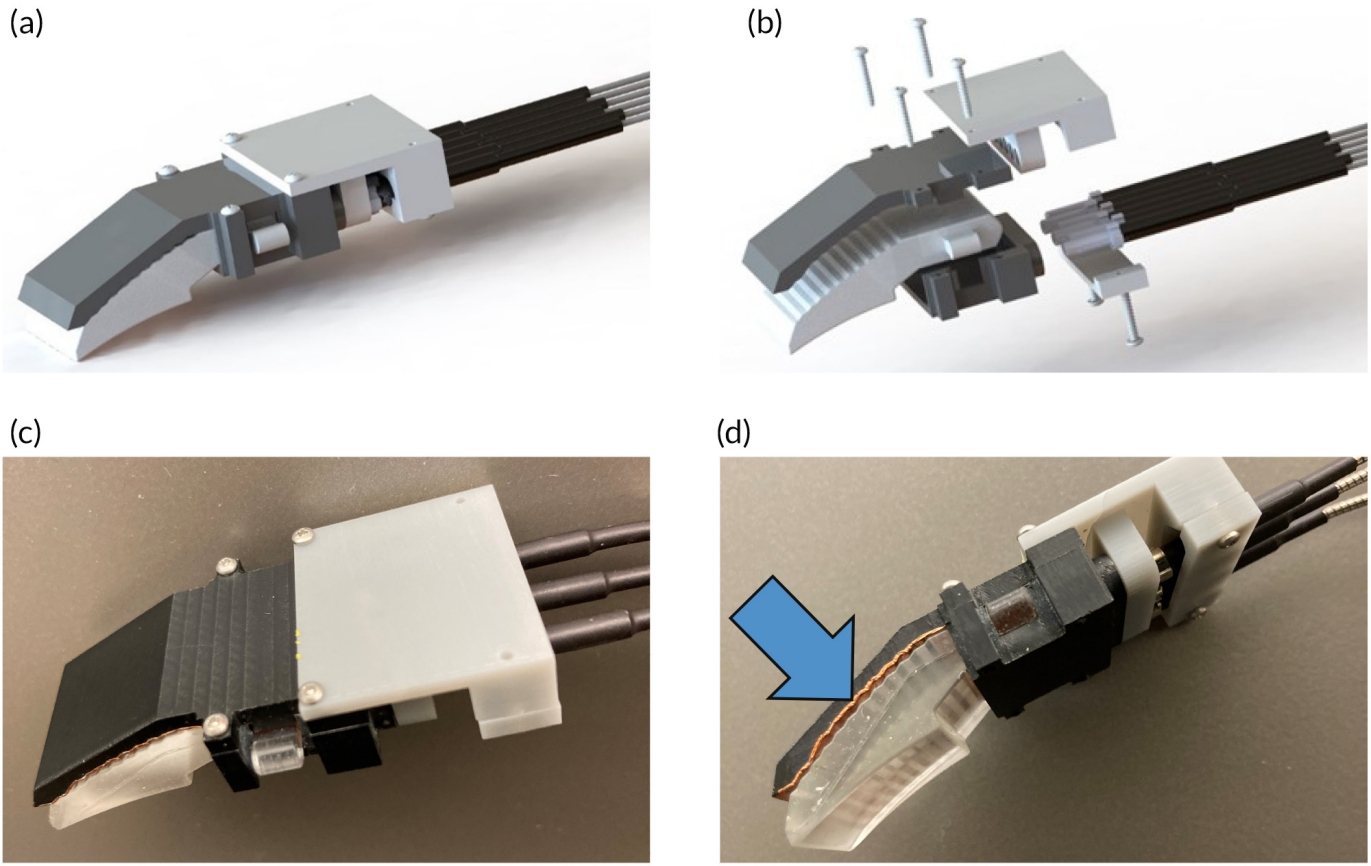
(a) CAD diagram of IDW. Reflective coating not shown. (b) Exploded‐view diagram of IDW. Reflective coating not shown. (c) Top down image of IDW. (d) Side profile of IDW. Blue arrow points to reflective copper tape lining the back of the extracting surface to reflect misdirected IRL back toward the skin surface. While not shown here to highlight the step‐wise extraction features of the IDW, the sides were also covered with reflective copper tape during testing.

The goal of this study was to generate IDWs with improved IRL delivery characteristics into the human body that (1) consider safety concerns by separating the IRL generation source and thus heat source from the human interface to limit unspecific heating at the IRL delivery point, (2) provide a more uniformly distributed IRL delivery over the entire area of the IDW, (3) allow direct contact of the IDW to the human skin by using a soft silicone that prevents pressure points, and (4) at the same time allow efficient IRL transmission compared with devices or IRL delivery approaches that deliver IRL through an air gap from, for example, an LED or a fiberoptic cable. The individual components of the IDW and their assembly are shown in Figure 1.

### 3.1 Virtual modeling

**FIGURE 2:**
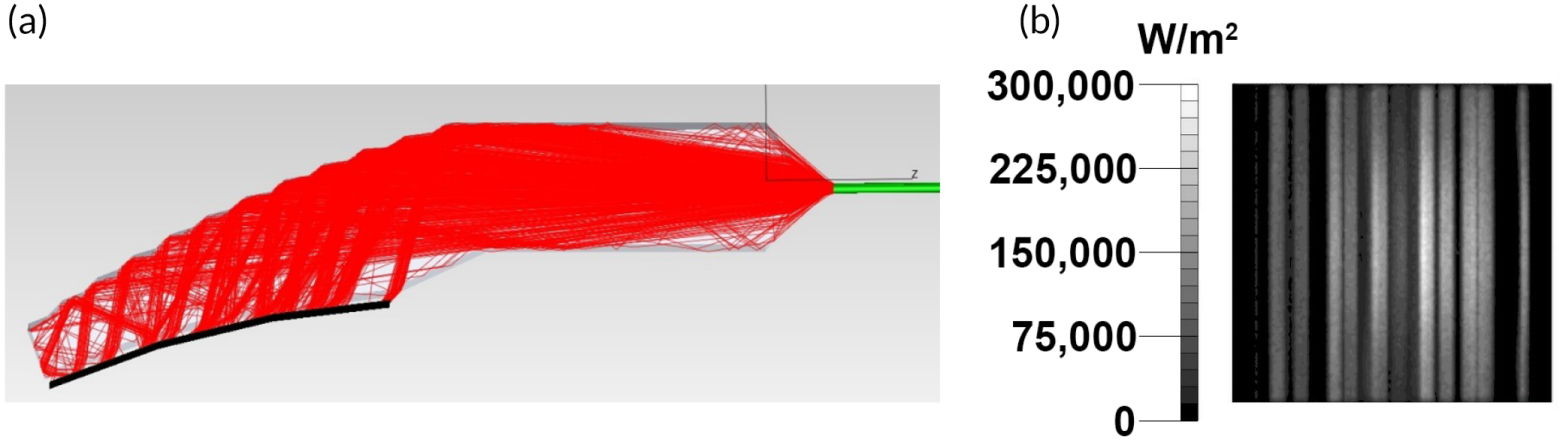
(a) Ray trace Monte Carlo simulation of IRL extracted by IDW. The black line denotes the lower edge of the IDW. (b) Output scan from ray trace Monte Carlo simulation. Simulation uses 23.693 W spread over 2,166,265 rays.

Ray trace Monte Carlo simulations demonstrated that an aspheric cylindrical collimator geometry with a 1.846 mm curvature radius positioned 3.5 mm from the IRL source and decentered by 0.3 ± 0.5 mm from the focal axis focused the maximum amount of IRL from the source to the extraction features (Figure 2a). The input angle of the IRL source was 0° and other details of the simulation are summarized in Table 1. The simulations also revealed that the waveguide maintained an extraction efficiency of ~80% covering the full emitting surface along the proximal‐to‐distal axis using an extraction feature step angle of 58° even when the emitting surface was curved to a 50 mm radial curve (Figure 2a). The ray traces showed a striated pattern of IRL output reflecting the patterning of steps along the IRL extracting surface (Figure 2b).

**TABLE 1:** Ray trace Monte Carlo simulation parameters

Input	Input angle	Material	A‐side surface	Extraction efficiency
Single light source	0°	Silicone	Mirror	79.0%

### 3.2 IDW form

The final IDW assembly form is represented in Figure 1. The final waveguide form contained an aspheric cylindrical collimating lens with a 1.846 mm radius of curvature that spanned the full width of the proximal end. A 20 mm channel was incorporated which allows the IRL to spread along the width axis before contacting the extraction features to improve uniformity of distribution along the right‐to‐left axis of the emitting surface. The IRL extracting surface was comprised of steps approximately 0.5 mm in height with a 58° slope angle spanning the length of the extracting surface. The emitting surface extended 6 mm beneath the bottom face of the channel to ensure the emitting surface would be superior to the IDW housing in the final assembly. The emitting surface was molded with a curvature to minimize the deformation necessary to form to a variety of head sizes and shapes within the patient population. Midway along the edge of the channel, two extruded bosses were molded to create an anchoring location to prevent the waveguide from moving out of positional tolerance with the housing and IRL input.

The clamshell housing contained two notches to accept the extruded boss anchor feature of the waveguide. The proximal face of the bottom housing contained six 2.5 mm dowel holes to accept three input ferrules each from the 750 nm splitter and three input ferrules from the 940 nm splitter. The fixture is mated with the ferrules and housing to further stabilize the IRL source ensuring the IRL source was offset with the center plane of the asymmetric lens by 0.3 ± 0.5 mm. The top housing and sides contained a light shield lined with reflective copper tape along the length of the extracting surface to reflect misdirected IRL back toward the skin surface (Figure 1d, blue arrow).

### 3.3 Waveguide extraction efficiency testing

Waveguide extraction efficiency testing was conducted to determine the ratio of total IRL power transferred through the emitting surface at the distal end to the total IRL power introduced through the collimating feature at the proximal end. Five waveguides were used to evaluate the extraction efficiency of 750 nm IRL, 940 nm IRL, and combined alternating inputs of 750 and 940 nm IRL. The extraction efficiency for combined wavelengths were calculated using Equations (1) and (2) for each condition with 1 W input for each individual wavelength (see Section 2). The testing was repeated doubling the input power/wavelength to 2 W to see if increasing the power resulted in a change in extraction efficiency. The average of extraction efficiency across all five waveguides is shown in Table 2.

**TABLE 2:** Average IDW IRL extraction efficiency comparing input IRL from the proximal end to IRL detected at emitting surface at the distal end (five independent IDWs were tested)

Light source input	Average extraction efficiency (*n* = 5)	SD (*n* = 5)
1 W 750 nm	79.20%	1%
2 W 750 nm	79.70%	1%
1 W 940 nm	79.10%	1%
2 W 940 nm	79.80%	1%
2 W Combined power	79.90%	1%
4 W Combined power	80.10%	0%

The waveguide extraction efficiencies from the physical model testing, ~80%, were nearly identical to the extraction efficiencies predicted by the virtual model ray trace simulations. None of the waveguides significantly deviated from the average extraction efficiency under any condition, showing no greater than a 1% SD.

### 3.4 Uniformity pixel analysis

**FIGURE 3:**
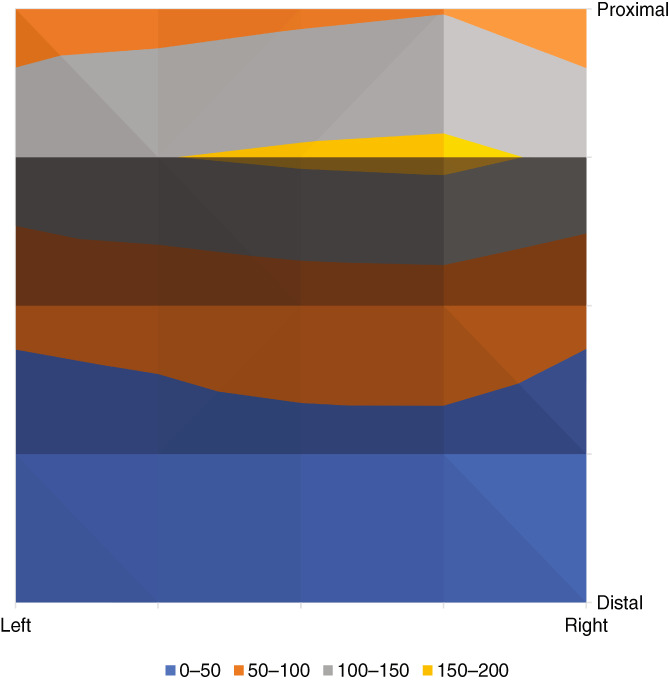
Surface plot map of average IRL intensity measured in milliwatts.

The distribution of output IRL power density from the emitting surface was measured through 4 × 4 mm “pixels” using a thermopile with photomask to determine the proportional disparity of IRL density from one location to the next across the emitting surface of the IDW (see Section 2). The emitting surfaces of five IDWs were measured pixel‐by‐pixel for a total of 25 locations per surface spanning the entire area. Table 3 shows a 5 × 5 cell matrix containing the average power output value of all five waveguides in each pixel location. The surface plot (Figure 3) shows a visual representation of IRL intensity through the emitting surface with the corresponding table values (Table 3) indicated by the plot vertices. The average surface percentage uniformity (SPU) value across all waveguides (calculated using Equation (4)) is 26% surface uniformity. The pixel values indicate a maximum power density near the center proximal end of the waveguide with a downward slope of diminishing intensity toward the distal end.

**TABLE 3:** Average IDW output IRL density map

Pixel location	(Left) 0–4 mm	4–8 mm	8–12 mm	12–16 mm	(Right) 16–20 mm
(Proximal) 0–4 mm	77.36	82.18	91.04	97.54	71.96
4–8 mm	134.4	149	156.4	160	142
8–12 mm	60.02	65.84	75.44	77.5	60.22
12–16 mm	26.1	31.48	36.62	36.7	25.08
(Distal) 16–20 mm	23.86	30.68	35.88	32.92	24.66

*Note*: Pixel power is presented in mW. Five independent IDWs were tested.

### 3.5 Emitting surface uniformity imaging

**FIGURE 4:**
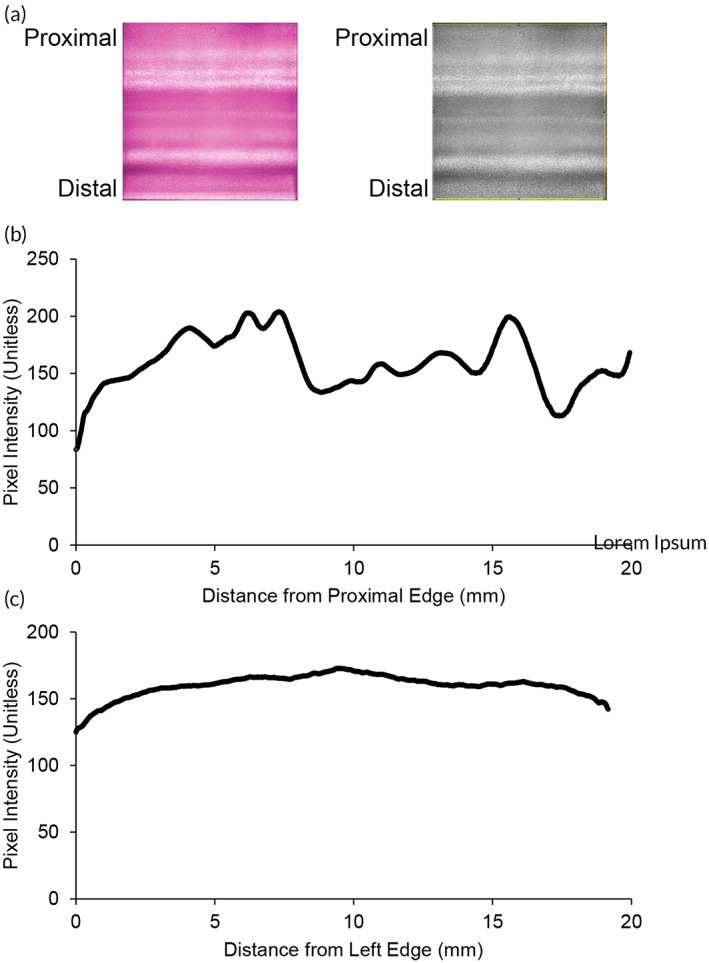
Emission uniformity profile of IDWs. (a) Representative raw image of IDW emission surface. (b) Average proximal‐distal distribution of IRL emitted from IDWs. (c) Average left–right distribution of IRL emitted from IDWs. Five independent IDWs were tested.

To show the uniformity of IRL emission from the IDW, the emitting surfaces of five IDWs were imaged using a Nikon camera converted to image IRL (Figure 4a). Each of the captured images were converted to 8‐bit gray‐scaled images using ImageJ. Next, the image scale was converted from pixels to millimeters. Pixel intensities were averaged along the left‐to‐right axis and plotted against the position along the distal‐to‐proximal axis (Figure 4b). The image was rotated by 90° and the process was repeated to average all of the pixel intensities along distal‐to‐proximal axis and plotted against the position along the left‐to‐right axis (Figure 4C).

### 3.6 Cadaver testing of the IDWs reveals improved IRL delivery efficiency

**FIGURE 5:**
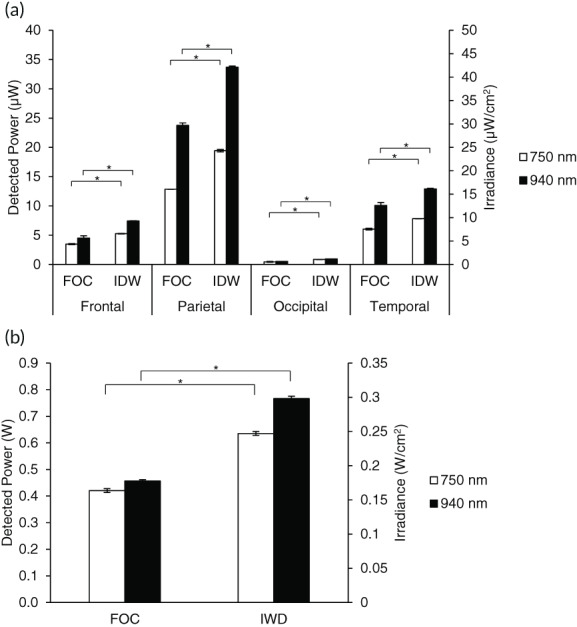
Human cadaver studies demonstrate superior IRL transmission of the IDW versus fiberoptic cable (FOC). (a) Power in microwatts and irradiance in microwatts per square centimeter of IRL detected after transmission through 4 cm of a whole, unfixed human cadaver head. Empty bars represent a 4 W input of 750 nm IRL. Filled bars represent a 4 W input of 940 nm IRL. (b) Power in watts and irradiance in watts per square centimeter of IRL detected after transmission through isolated cadaveric skin. Empty bars represent a 1 W input of 750 nm IRL. Filled bars represent a 1 W input of 940 nm IRL. Skin sample was 0.37 cm thick. The IDW was in direct contact with the skin, whereas the fiberoptic cable was positioned at 2 and 3 cm distance from the skin in a and b, respectively. Statistical significance was determined using a two‐tailed Student's *t* test. *N* = 5; *p < 0.05. Error bars represent SD.

Donor was an 80‐year‐old, Caucasian female with a recorded weight of 40.8 kg and height of 160.0 cm. IRL transmission measurements were performed 25 days post‐mortem. Similar to our previous work^25^ examining IRL transmission through whole and unfixed cadavers, four lobes of the brain were evaluated as an IRL delivery point in order to compare the difference in IRL intensity delivered to 4 cm in the brain by the fiberoptic cable versus the waveguide (Figure 5a). For the frontal lobe, the IDW delivered 51% and 65% more IRL than the fiberoptic for 750 and 940 nm IRL, respectively. The parietal lobe saw similar levels, with the IDW delivering 51% and 42% more IRL than the laser for 750 and 940 nm IRL, respectively. The occipital lobe experienced almost a doubling in the detected power of IRL with 95% and 81% more IRL than the laser for 750 and 940 nm IRL, respectively. Meanwhile, the temporal lobe experienced a smaller increase in IRL delivered with the IDW over the fiberoptic with a 30% and 28% increase for 750 and 940 nm IRL, respectively. Overall, for each lobe of the brain, the waveguide delivered significantly more IRL into the brain than the fiberoptic cable.

IRL has been proposed as a therapeutic for conditions outside of the brain. No matter the biological target, IRL will first need to penetrate the skin. Therefore, skin samples were taken from the cadaver in order to compare IRL transmission by the fiberoptic cable to the IDW. For the isolated skin transmission measurements (Figure 5b) the waveguide delivered 51% and 68% more IRL than the fiberoptic cable for 750 and 940 nm IRL, respectively.

## 4 DISCUSSION

Here, we described the design and testing of our IDWs which use waveguide technology to efficiently deliver COX‐inhibitory IRL from the laser source into the human head to treat conditions such as stroke. The IDWs have several design aspects that protect patient safety from the high‐intensity IRL while also facilitating transmission of IRL into the head. The IDWs are constructed of low‐durometer silicone, which conforms to the patient's scalp and has an index of refraction in the median range of skin (∼1.41). The direct contact allows IRL to immediately exit the waveguide and enter the skin, which omits the need for emitter surface‐to‐air and air‐to‐tissue transition layers and likely explains the increased IRL transmission seen with our cadaveric model. The low durometer silicone prevents pressure points, allowing for longer treatment durations while reducing the risk of scalp sores to the patient. Due to the extraction step design of the IDWs, IRL is emitted in a uniform distribution, which prevents “hot spots” which could burn the skin. Additionally, by coating the external surface with reflective material, IRL that is initially reflected off of the skin can be redirected back toward the treatment area.

The design of the IDW was guided by ray trace Monte Carlo simulation for maximal extraction efficiency. After construction, an extraction efficiency of IRL output to IRL input was ~80%, nearly identical to what was predicted with the Monte Carlo simulation. Measurements of the actual output surface reveal that more IRL tends to be emitted from the proximal side of the IDWs compared with the distal site. There was little variation in emission when comparing the right and left sides of the IDWs.

We have previously worked with fresh, unfixed cadavers to quantitate the transmission of IRL to a depth of 4 cm into the human head. Here, we compared the transmission of IRL emitted via a fiberoptic versus our IDW. The fiberoptic emits IRL as a cone. The fiberoptic was positioned such that the surface area of its emission profile was 4 cm^2^, the same as the IDW. In this manner, the fiberoptic and IDW had the same input power and irradiance at the surface of the skin, delivered in a surface area of similar size. Using the same output IRL power intensities, the IDWs showed transmission of IRL to a depth of 4 cm that was up to 95% and 81% increased for 750 nm and 940 nm, respectively, compared with the fiberoptic alone. Consistently, the IDWs showed transmission of IRL through 0.37 cm isolated skin that was up to 51% and 68% increased for 750 and 940 nm, respectively, compared with the fiberoptic cable. As we discussed in detail^25^ the overall lower IRL penetration at the occipital position is due to cadaver positioning (face up), leading to formation of coagulated blood pools that form between the skull and the skin at this site. As the pooled blood absorbs IRL, the transmission values for the occipital lobe are an underestimation of what would be achieved when treating a living patient. Experimental IRL transmission measurements were carried out quickly (<10 s), and no measurable change in temperature was detected. However, we predict that, even when treating a living organism for several hours, the circulatory system and the body's natural thermoregulatory systems would act as a heat sink, preventing localized heating.

Overall, the IDWs offer a scalable solution for therapeutic IRL delivery that outperforms laser delivery. The flexibility and size of the IDWs allow for them to be comfortably integrated into a cap to be easily applied after a stroke and allow for full coverage of the entirety of the head. For future iterations of the IDWs, we plan on further improving both extraction efficiency as well as proximal‐distal emission uniformity. Use of waveguides has multiple advantages. Because the IRL is distributed over a larger surface area (4 cm^2^) instead of a small circle emitted by the fiberoptic (about 1 mm) the risk of heating up or burning the skin is mitigated. Furthermore, because the waveguide is in direct contact with the skin, the air‐to‐skin transition of the IRL otherwise delivered through the fiberoptic cable at a 2–3 cm distance is omitted. This likely explains the higher IRL delivery efficiency, ranging from a 28% to 95% increase for the IDW compared with the fiberoptic cable, depending on the IRL delivery area of the head. In addition, IRL that is back reflected into the waveguide has a higher chance of being back reflected into the head due to the reflective coating of the waveguide further augmenting IRL delivery efficiency into the brain. Overall, the IDW offers a scalable solution for enhanced tissue delivery of therapeutic IRL.

## AUTHOR CONTRIBUTIONS

**Paul T. Morse:** Data curation (equal); formal analysis (equal); investigation (equal); methodology (equal); validation (equal); visualization (equal); writing – original draft (equal); writing – review and editing (lead). **Samuel Tuck:** Conceptualization (equal); data curation (equal); formal analysis (equal); investigation (equal); methodology (equal); software (lead); validation (equal); visualization (equal); writing – original draft (lead). **Mike Kerns:** Investigation (supporting); methodology (supporting); project administration (supporting); resources (equal); software (equal). **Dennis J. Goebel:** Investigation (equal); methodology (equal); resources (equal); supervision (equal). **Junmei Wan:** Investigation (equal); methodology (equal). **Tom Waddell:** Conceptualization (equal); investigation (equal); methodology (equal); project administration (equal); supervision (equal); writing – review and editing (equal). **Joseph M. Wider:** Investigation (supporting); methodology (supporting). **Charlotte L. Hüttemann:** Investigation (supporting). **Moh H. Malek:** Project administration (supporting); supervision (supporting). **Icksoo Lee:** Project administration (supporting); supervision (supporting); writing – review and editing (equal). **Thomas H. Sanderson:** Conceptualization (equal); funding acquisition (equal); investigation (equal); project administration (equal); resources (equal); supervision (equal); writing – review and editing (equal). **Maik Hüttemann:** Conceptualization (equal); funding acquisition (lead); investigation (equal); methodology (equal); project administration (lead); resources (lead); supervision (lead); writing – original draft (equal); writing – review and editing (equal).

## CONFLICT OF INTEREST

Maik Hüttemann and Thomas H. Sanderson are co‐founders of Mitovation, Inc. that develops IRL therapy for ischemia/reperfusion injury applications. Samuel Tuck served as systems engineer and Tom Waddell serves as COO of Mitovation, Inc. All other authors declare no potential conflict of interest.

### PEER REVIEW

The peer review history for this article is available at https://publons.com/publon/10.1002/btm2.10496.

## ETHICS STATEMENT

In accordance with the Uniform Anatomical Gift Act of Michigan (Act. No. 368, Public Acts of 1978, Article 10), donor consent for educational and research purposes was obtained prior to death.

## Data Availability

The data that support the findings of this study are available from the corresponding author upon reasonable request.

## References

[1] Johnson CO , Nguyen M , Roth GA , et al. Global, regional, and national burden of stroke, 1990–2016: a systematic analysis for the global burden of disease study 2016. Lancet Neurol. 2019;18(5):439‐458. 30871944 10.1016/S1474-4422(19)30034-1 PMC6494974

[2] Tsao CW , Aday AW , Almarzooq ZI , et al. Heart disease and stroke statistics‐2022 update: a report from the American Heart Association. Circulation. 2022;145(8):e153‐e639. 35078371 10.1161/CIR.0000000000001052

[3] Lo EH , Moskowitz MA , Jacobs TP . Exciting, radical, suicidal: how brain cells die after stroke. Stroke. 2005;36(2):189‐192. 15637315 10.1161/01.STR.0000153069.96296.fd

[4] Kalpage HA , Wan J , Morse PT , Lee I , Hüttemann M . Brain‐specific serine‐47 modification of cytochrome *c* regulates cytochrome *c* oxidase activity attenuating ROS production and cell death: implications for ischemia/reperfusion injury and Akt signaling. Cells. 2020;9(8):1‐18. 10.3390/cells9081843 PMC7465522 32781572

[5] Chouchani ET , Pell VR , Gaude E , et al. Ischaemic accumulation of succinate controls reperfusion injury through mitochondrial ROS. Nature. 2014;515(7527):431‐435. 25383517 10.1038/nature13909 PMC4255242

[6] Kalpage HA , Wan J , Morse PT , et al. Cytochrome *c* phosphorylation: control of mitochondrial electron transport chain flux and apoptosis. Int J Biochem Cell Biol. 2020;121:105704. 32023432 10.1016/j.biocel.2020.105704 PMC7044036

[7] Hüttemann M , Helling S , Sanderson TH , et al. Regulation of mitochondrial respiration and apoptosis through cell signaling: cytochrome *c* oxidase and cytochrome *c* in ischemia/reperfusion injury and inflammation. Biochim Biophys Acta. 2012;1817(4):598‐609. 21771582 10.1016/j.bbabio.2011.07.001 PMC3229836

[8] A randomized trial of tirilazad mesylate in patients with acute stroke (RANTTAS). The RANTTAS investigators. Stroke. 1996;27(9):1453‐1458. 8784112 10.1161/01.str.27.9.1453

[9] van der Worp HB , de Haan P , Morrema E , Kalkman CJ . Methodological quality of animal studies on neuroprotection in focal cerebral ischaemia. J Neurol. 2005;252(9):1108‐1114. 16170651 10.1007/s00415-005-0802-3

[10] Hamblin MR . Photobiomodulation for traumatic brain injury and stroke. J Neurosci Res. 2018;96(4):731‐743. 29131369 10.1002/jnr.24190 PMC5803455

[11] Karu TI , Afanas'eva NI . Cytochrome c oxidase as the primary photoacceptor upon laser exposure of cultured cells to visible and near IR‐range light. Dokl Akad Nauk. 1995;342(5):693‐695. 7670387

[12] Karu T . Primary and secondary mechanisms of action of visible to near‐IR radiation on cells. J Photochem Photobiol B. 1999;49(1):1‐17. 10365442 10.1016/S1011-1344(98)00219-X

[13] Wharton DC , Tzagoloff A . Studies on the electron transfer system. Lvii. The near infrared absorption band of cytochrome oxidase. J Biol Chem. 1964;239:2036‐2041. 14213394

[14] Chen AC , Arany PR , Huang YY , et al. Low‐level laser therapy activates NF‐kB via generation of reactive oxygen species in mouse embryonic fibroblasts. PLoS One. 2011;6(7):e22453. 21814580 10.1371/journal.pone.0022453 PMC3141042

[15] Sanderson TH , Wider JM , Lee I , et al. Inhibitory modulation of cytochrome c oxidase activity with specific near‐infrared light wavelengths attenuates brain ischemia/reperfusion injury. Sci Rep. 2018;8(1):3481. 29472564 10.1038/s41598-018-21869-x PMC5823933

[16] Strubakos CD , Malik M , Wider JM , et al. Non‐invasive treatment with near‐infrared light: a novel mechanisms‐based strategy that evokes sustained reduction in brain injury after stroke. J Cereb Blood Flow Metab. 2020;40(4):833‐844. 31112450 10.1177/0271678X19845149 PMC7168789

[17] Jacques SL . Optical properties of biological tissues: a review. Phys Med Biol. 2013;58(11):R37‐R61. 23666068 10.1088/0031-9155/58/11/R37

[18] Esnouf A , Wright PA , Moore JC , Ahmed S . Depth of penetration of an 850nm wavelength low level laser in human skin. Acupunct Electrother Res. 2007;32(1‐2):81‐86. 18077939 10.3727/036012907815844165

[19] Xu Z , Xie Q , Tan Z , Wu Q , Chen Y . Heat‐resistant optical waveguides using new silicone‐based polymers. In First Joint Symposium on Opto‐ and Microelectronic Devices and Circuits; 2000:138–141.

[20] Beebe JM , Swatowski BW , Weidner WK , et al. Semiquantitative atomic force microscopy‐infrared spectroscopy analysis of chemical gradients in silicone optical waveguides. ACS Appl Mater Interfaces. 2020;12(9):11287‐11295. 32049488 10.1021/acsami.0c00350

[21] Kocheril PA , Lenz KD , Mascareñas DDL , Morales‐Garcia JE , Anderson AS , Mukundan H . Portable waveguide‐based optical biosensor. Biosensors. 2022;12:4. 10.3390/bios12040195 PMC9025188 35448255

[22] Mittal V , Nedeljkovic M , Carpenter LG , et al. Waveguide absorption spectroscopy of bovine serum albumin in the mid‐infrared fingerprint region. ACS Sens. 2019;4(7):1749‐1753. 31264410 10.1021/acssensors.9b00215

[23] Zhang H , Zhao H , Zhao X , et al. Biocompatible light guide‐assisted wearable devices for enhanced UV light delivery in deep skin. Adv Funct Mater. 2021;31(23):2100576.

[24] Heiskanen V , Hamblin MR . Photobiomodulation: lasers vs. light emitting diodes? Photochem Photobiol Sci. 2018;17(8):1003‐1017. 30044464 10.1039/c8pp00176f PMC6091542

[25] Morse PT , Goebel DJ , Wan J , et al. Cytochrome c oxidase‐modulatory near‐infrared light penetration into the human brain: implications for the noninvasive treatment of ischemia/reperfusion injury. IUBMB Life. 2021;73(3):554‐567. 33166061 10.1002/iub.2405 PMC8819601

